# Metal-Based Antibacterial and Antifungal Agents: Synthesis, Characterization, and In Vitro Biological Evaluation of Co(II), Cu(II), Ni(II), and Zn(II) Complexes With Amino Acid-Derived Compounds

**DOI:** 10.1155/BCA/2006/83131

**Published:** 2006-12-20

**Authors:** Zahid H. Chohan, M. Arif, Muhammad A. Akhtar, Claudiu T. Supuran

**Affiliations:** ^1^Department of Chemistry, Bahauddin Zakariya University, Multan 60800, Pakistan; ^2^Laboratorio di Chimica Bioinorganica, Dipartimento di Chimica, Polo Scientifico e Tecnologico, Università degli Studi di Firenze, Rm 188, Via della Lastruccia 3, 50019 Sesto Fiorentino, Florence, Italy

## Abstract

A series of antibacterial and antifungal amino acid-derived compounds and their cobalt(II), copper(II), nickel(II), and zinc(II) metal complexes have been synthesized and characterized by their elemental analyses, molar conductances, magnetic moments, and IR, and electronic spectral measurements. Ligands (L_1_)−(L_5_) were derived by condensation of *β*-diketones with glycine, phenylalanine, valine, and histidine and act as bidentate towards metal ions (cobalt, copper, nickel, and zinc) via the azomethine-N and deprotonated-O of the respective amino acid. The stoichiometric reaction between the metal(II) ion and synthesized ligands in molar ratio of M : L (1 : 1) resulted in the formation of the metal complexes of type [M(L)(H_2_O)_4_]Cl (where M = Co(II), Cu(II), and Zn(II)) and of M : L (1 : 2) of type [M(L)_2_(H_2_O)_2_] (where M = Co(II), Cu(II), Ni(II), and Zn(II)). The magnetic moment data suggested for the complexes to have an octahedral geometry around the central metal atom. The electronic spectral data also supported the same octahedral geometry of the complexes. Elemental analyses and NMR spectral data of the ligands and their metal(II) complexes agree with their proposed structures. The synthesized ligands, along with their metal(II) complexes, were screened for their in vitro antibacterial activity against four Gram-negative (*Escherichia coli, Shigella flexeneri, Pseudomonas aeruginosa, and Salmonella typhi*) and two Gram-positive (*Bacillus subtilis* and *Staphylococcus aureus*) bacterial strains and for in vitro antifungal activity against *Trichophyton longifusus, Candida albicans, Aspergillus flavus, Microsporum canis, Fusarium solani, and Candida glaberata*. The results of these studies show the metal(II) complexes to be more antibacterial/antifungal against one or more species as compared to the uncomplexed ligands. The brine shrimp bioassay was also carried out to study their in vitro cytotoxic properties. Five compounds, (3), (7), (10), (11), and (22), displayed potent cytotoxic activity as LD_50_ = 8.974 × 10^−4^, 7.022 × 10^−4^, 8.839 × 10^−4^, 7.133 × 10^−4^, and 9.725 × 10^−4^ M/mL, respectively, against *Artemia salina*.

## INTRODUCTION

**Figure 1:**
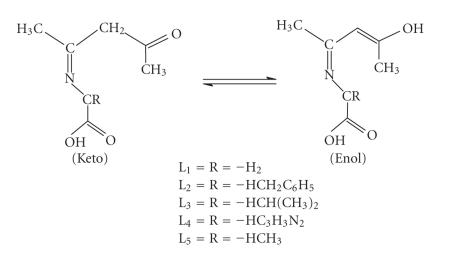
Proposed structure of the ligands (L_1_)–(L_5_).

We have already drawn attention [1–5] to the strong relationship between metals or their complexes, and antibacterial [6–12], antitumour [13–15], and anticancer [16, 17] activities. A number of in vivo studies have indicated [18–20] that biologically active compounds become more bacteriostatic and carcinostatic upon chelation. Such interaction of transition-metal ions with amino acids and peptides is of immense biological importance [21–23]. It has been reported [24–28] that metal complexes of amino acid Schiff bases with transition metals possess anticarcinogenic activity. Various tumors tend to have poor blood supplies, and therefore amino acids have been effectively used to direct nitrogen mustards into the cancer cells. For example, phenylalanine mustard is used in controlling malignant myeloma [29] and Burkett's lymphoma [30], and similarly sarcolysine [31] is used to treat wide range of tumors. Indeed, certain tumors and cancer cells are unable to produce all the amino acids synthesized by the normal cells. Therefore, these cells require an external supply of such essential amino acids to pass on to the cancer cells by the blood stream. In the recent past, a number of studies have highlighted the use of acetylacetone in various significant applications [32–37]. In the present studies, ligands (L_1_)–(L_5_) (Figure 1) were obtained by the condensation reaction between amino acids (glycine, phenylalanine, alanine, valine, or histidine) and acetylacetone with this hope that it may provide us valuable theoretical information for exploring metal-based bacteriostatic and/or carcinostatic pharmaceuticals with high efficacy and low toxicity. In this effort, we have also introduced an azomethine (−C=N) linkage with the concern that it may permit a notable variety in the remarkable chemistry and behavior of such compounds. The synthesized amino acid-derived compounds (L_1_)–(L_5_) have been exposed to act as bidentate towards divalent metal atoms solely through the azomethine-N and carboxylato groups forming a stable 5-membered chelate ring system. The metal(II) complexes, (1)–(40) of the types [M(L)(H_2_O)_4_] and [M(L)_2_(H_2_O)_2_]Cl (where M = Co(II), Cu(II), Ni(II), and Zn(II) and L = amino acid-derived ligands (L_1_)–(L_5_)) were formed by a stoichiometric ratio of M : L as (1 : 2) and (1 : 1), respectively. These two different stoichiometric ratios of the ligand incorporated with the metal ion were used in order to study the effect of the presence of one or two ligands, respectively, on the biological activity. All these compounds have been characterized by their IR, NMR, molar conductance, magnetic moment, and elemental analyses. The IR of the ligands and their corresponding metal(II) complexes are in agreement with the proposed structures. The magnetic moment and electronic spectral data suggest for all the complexes to have an octahedral geometry. Elemental analyses and NMR spectral data of the ligands and their metal(II) complexes also agree with the structures as anticipated. All these ligands along with their metal(II) complexes were screened for their in vitro antibacterial activity against four Gram-negative (*E coli, S flexenari, P aeruginosa, and S typhi*) and two Gram-positive (*B subtilis* and *S aureus*) bacterial strains and for in vitro antifungal activity against *T longifusus, C albicans, A flavus, M canis, F solani, and C glaberata*. These compounds have shown varied antibacterial and antifungal activities against one or more bacterial/fungal strains and this activity enhanced on coordination/chelation. The reported compounds are not only good candidates as antibacterial and antifungal agents, but also are a promising addition of new class of compounds as the metal-based drugs.

## EXPERIMENTAL

### Material and methods

Solvents used were analytical grades; all metal(II) were used as chloride salts. IR spectra were recorded on the Philips Analytical PU 9800 FTIR spectrophotometer. NMR spectra were recorded on Perkin-Elmer 283B spectrometer. UV-visible spectra were obtained in DMF on a Hitachi U-2000 double-beam spectrophotometer. C, H, and N analyses, conductance and magnetic measurements were carried out on solid compounds using the respective instruments. Melting points were recorded on a Gallenkamp apparatus and are not corrected. The complexes were analyzed for their metal contents by EDTA titration [38]. Antibacterial and antifungal screening was done at HEJ Research Institute of Chemistry, International Center for Chemical Sciences, University of Karachi, Pakistan.

### Preparation of Schiff-bases (L_1_)–(L_5_)

Acetylacetone (20 mmol) in ethanol (10 mL) was added to a stirred solution of the amino acid (20 mmol) in water (30 mL). The mixture was refluxed for 4–6 hours during which the color of the solution turned to yellow-orange. The completion of reaction was monitored through TLC. After completion of the reaction, it was cooled to afford a solid product. The solid residue was filtered, washed with ethanol, then with ether, and dried. Crystallization from a mixture of ethanol-propanol (60 : 40) afforded the desired ligands. The same method was applied for the preparation of all other ligands by using the corresponding amino acids and/or acetylacetone, working in the same conditions with their respective molar ratio.

### {[(3-Hydroxy-1-methylbutyl)-2-en-1-ylidene] amino}acetic acid (L_1_)

Yield 52%; mp 294°C; IR (KBr, cm^−1^): 3444 (OH), 3015 (C=C), 1700 (COOH), 1635 (azomethine, HC=N); ^1^H NMR (DMSO-d_6_, *δ*, ppm): 1.85 (s, 6H, CH_3_), 2.83 (t, 2H, CH_2_), 5.18 (t, 1H, CH), 6.94 (s, 1H, azomethine), 10.27 (s, 1H, OH), 11.29 (s, 1H, COOH). Anal. Calcd. for C_7_H_11_NO_3_ (157.0): C, 53.50; H, 7.01; N, 8.92. Found: C, 53.32; H, 7.41; N, 8.86%. ^1^H NMR of Zn(II) complex (DMSO-d_6_, *δ*, ppm): 2.08 (s, 6H, CH_3_), 2.98 (t, 2H, CH_2_), 5.37 (t, 1H, CH), 7.48 (s, 1H, azomethine), 10.58 (s, 1H, OH), 11.36 (s, 4H, OH_2_).

### {[2-(3-Hydroxy-1-methylbutyl)-2-en-1-ylidene]amino}-3-phenylpropanoic acid (L_2_)

Yield 56%; mp 242°C; IR (KBr, cm^−1^): 3444 (OH), 3049 (C=C), 1703 (COOH), 1635 (azomethine, C=N); ^1^H NMR (DMSO-d_6_, *δ*, ppm): ^1^H NMR (DMSO-d_6_, *δ*, ppm): 1.75 (s, 6H, CH_3_), 2.53 (t, 2H, CH_2_), 3.18 (t, 1H, CH_2_), 3.73 (t, 2H, CH_2_), 6.67 (s, 1H, azomethine), 7.16–7.79 (m, 5H, Ph), 10.27 (s, 1H, OH), 11.29 (s, 1H, COOH). Anal. Calcd. for C_14_H_19_NO_2_ (233.0): C, 68.02; H, 6.88; N, 5.67. Found: C, 68.33; H, 7.15; N, 5.83%. ^1^H NMR of Zn(II) complex (DMSO-d_6_, *δ*, ppm): 1.97 (s, 6H, CH_3_), 2.86 (t, 2H, CH_2_), 3.41 (t, 1H, CH_2_), 3.96 (t, 2H, CH_2_), 7.51 (s, 1H, azomethine), 7.36–7.93 (m, 5H, Ph), 10.58 (s, 1H, OH), 11.36 (s, 4H, OH_2_).

### {[2-(3-Hydroxy-1-methylbutyl)-2-en-1-ylidene]amino}-3-methylbutanoic acid (L_3_)

Yield 54%; mp 210°C; IR (KBr, cm^−1^): 3444 (OH), 3049 (C=C), 1708 (COOH), 1635 (azomethine, C=N); ^1^H NMR (DMSO-d_6_, *δ*, ppm): 1.88 (s, 12H, CH_3_), 3.16 (t, 1H, CH), 3.73 (t, 1H, CH), 5.52 (t, 1H, CH), 10.27 (s, 1H, OH), 11.29 (s, 1H, COOH). Anal. Calcd. for C_10_H_17_NO_3_ (199.0): C, 60.30; H, 8.54; N, 7.04. Found: C, 60.64; H, 8.37; N, 7.46%. ^1^H NMR of Zn(II) complex (DMSO-d_6_, *δ*, ppm): 2.03 (s, 12H, CH_3_), 3.37 (t, 1H, CH), 3.96 (t, 1H, CH), 5.87 (t, 1H, CH), 10.56 (s, 1H, OH), 11.36 (s, 4H, OH_2_).

### {[2-(3-Hydroxy-1-methylbutyl)-2-en-1-ylidene]amino}-3-(imidazol-4-yl) propanoic acid (L_4_)

Yield 51%; mp 194°C; IR (KBr, cm^−1^): 3444 (OH), 3045 (C=C), 1705 (COOH), 1635 (azomethine, C=N); ^1^H NMR (DMSO-d_6_, *δ*, ppm): ^1^H NMR (DMSO-d_6_, *δ*, ppm): 1.75 (s, 6H, CH_3_), 3.36 (t, 1H, CH), 3.78 (s, 1H, CH), 7.96 (s, 1H, imidazol), 8.26 (d, 1H, imidazol), 10.27 (s, 1H, OH), 10.84 (s, 1H, NH), 11.29 (s, 1H, COOH). Anal. Calcd. for C_10_H_13_N_3_O_3_ (223.0): C, 55.23; H, 7.11; N, 17.53. Found: C, 55.53; H, 7.38; N, 17.26%; ^1^H NMR of Zn(II) complex (DMSO-d_6_, *δ*, ppm): 2.07 (s, 6H, CH_3_), 3.58 (t, 1H, CH), 3.94 (s, 1H, CH), 8.25 (s, 1H, imidazol), 8.47 (dd, 1H, imidazol), 10.58 (s, 1H, OH), 11.13 (s, 1H, NH), 11.36 (s, 4H, OH_2_).

### {[2-(3-Hydroxy-1-methylbutyl)-2-en-1-ylidene]amino}propanoic acid (L_5_)

Yield 53%; mp 160°C; IR (KBr, cm^−1^): 3444 (OH), 3018 (C=C), 1700 (COOH), 1635 (azomethine, C=N); ^1^H NMR (DMSO-d_6_, *δ*, ppm): 1.85 (s, 9H, CH_3_), 5.18 (t, 1H, CH), 5.34 (t, 1H, CH), 10.27 (s, 1H, OH), 11.29 (s, 1H, COOH). Anal. Calcd. for C_8_H_13_NO_3_ (171.0): C, 47.76; H, 7.46; N, 20.90. Found: C, 47.57; H, 7.28; N, 20.77%. ^1^H NMR of Zn(II) complex (DMSO-d_6_, *δ*, ppm): 2.12 (s, 9H, CH_3_), 5.41 (t, 1H, CH), 5.63 (t, 1H, CH), 10.58 (s, 1H, OH), 11.36 (s, 4H, OH_2_).

### Preparation of metal(II) complexes

For the preparation of metal(II) complexes, a solution (30 mL) of the corresponding ligand in hot methanol was added to a stirred solution of metal(II) chloride in ethanol (25 mL) having a required molar ratio of M : L (1 : 1 and 1 : 2). The mixture was refluxed for 3 hours and then cooled to room temperature which solidified on cooling. The solid thus obtained was filtered, washed with methanol/ethanol and ether, and finally dried in air to afford the desired product. Crystallization from aqueous/ethanol (40 : 60) gave the expected metal complex.

## BIOLOGICAL ACTIVITY

### Antibacterial bioassay (in vitro)

All the synthesized ligands (L_1_)–(L_5_) and their corresponding metal(II) complexes (1)–(20) were screened in vitro for their antibacterial activity against four Gram-negative (*E coli, S flexenari, P aeruginosa, and S typhi*) and two Gram-positive (*B subtilis* and *S aureus*) bacterial strains using agar-well diffusion method [39]. Two to eight hours old bacterial inoculums containing approximately 10^4^–10^6^ colony forming units (CFU)/mL were used in these assays. The wells were dug in the media with the help of a sterile metallic borer with centers at least 24 mm. Recommended concentration (100 *μ*l) of the test sample (1 mg/mL in DMSO) was introduced in the respective wells. Other wells supplemented with DMSO and reference antibacterial drug, imipenum served as negative and positive controls, respectively. The plates were incubated immediately at 37°C for 20 hours. Activity was determined by measuring the diameter of zones showing complete inhibition (mm). Growth inhibition was compared [40] with the standard drug. In order to clarify any participating role of DMSO in the biological screening, separate studies were carried out with the solutions alone of DMSO and they showed no activity against any bacterial strains.

### Antifungal activity (in vitro)

Antifungal activities of all compounds were studied against six fungal cultures, *T longifusus, C albicans, A flavus, M canis, F solani*, and *C glaberata*. Sabouraud dextrose agar (Oxoid, Hampshire, England) was seeded with 10^5^ (cfu) mL^−1^ fungal spore suspensions and was transferred to petri plates. Discs soaked in 20 mL (10 *μ*g/mL in DMSO) of all compounds were placed at different positions on the agar surface. The plates were incubated at 32°C for seven days. The results were recorded as zones of inhibition in mm and were compared with standard drugs Miconazole and Amphotericin B.

### Minimum inhibitory concentration (MIC)

Compounds containing antibacterial activity over 80% were selected for minimum inhibitory concentration (MIC) studies (Table 5). The minimum inhibitory concentration was determined using the disc diffusion technique [39] by preparing discs containing 10, 25, 50, and 100 *μ*g/mL of the compounds and applying the protocol.

### Cytotoxicity (in vitro)

Brine shrimp (*Artemia salina* leach) eggs were hatched in a shallow rectangular plastic dish (22×32 cm), filled with artificial seawater, which was prepared [24] with commercial salt mixture and double distilled water. An unequal partition was made in the plastic dish with the help of a perforated device. Approximately 50 mg of eggs were sprinkled into the large compartment, which was darkened while the matter compartment was opened to ordinary light. After two days, nauplii were collected by a pipette from the lighted side. A sample of the test compound was prepared by dissolving 20 mg of each compound in 2 mL of DMF. From this stock solutions, 500, 50, and 5 *μ*g/mL were transferred to 9 vials (three for each dilution were used for each test sample and LD_50_ is the mean of three values) and one vial was kept as control having 2 mL of DMF only. The solvent was allowed to evaporate overnight. After two days, when shrimp larvae were ready, 1 mL of seawater and 10 shrimps were added to each vial (30 shrimps/dilution) and the volume was adjusted with seawater to 5 mL per vial. After 24 hours, the numbers of survivors were counted. Data were analyzed by Finney computer program to determine the LD_50_ values [41].

## RESULT AND DISCUSSION

### Physicochemical properties of obtained compounds

**Figure 2:**
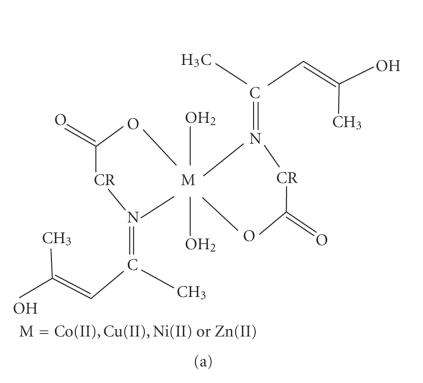

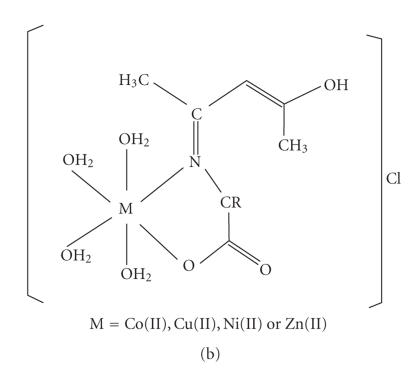
Proposed structures of the metal(II) complexes (1)–(40).

The ligands (L_1_)–(L_5_) were prepared by refluxing an appropriate amount of respective amino acid with the corresponding acetylacetone in ethanol. The structures of the synthesized ligands were established with the help of their IR, NMR, and microanalytical data. All metal(II) complexes (1)–(40) of these ligands were prepared by using the respective metal salts as chloride with the corresponding ligands in two different molar ratios of metal : ligand as 1 : 2 and 1 : 1. All these complexes are intensively colored air and moisture stable amorphous solids which decompose without melting. They are insoluble in common organic solvents and only soluble in water, DMF, and DMSO. Molar conductance values of the soluble complexes in DMF (10^−3^ M solution at 25°C) indicated that complexes having molar ratio of metal : ligand as 1 : 2 have lower values (26–35 Ohm^−1^ cm^−2^ mol^−1^) indicating that they are all nonelectrolytic in nature. However, the complexes having molar ratio of metal : ligand as 1 : 1 showed higher values (122–128 Ohm^−1^ cm^−2^ mol^−1^) indicating them as electrolytic [42]. The elemental analyses data (Table 1) agree well with the proposed formulae for the ligands and also confirmed the [M(L)_2_(OH_2_)_2_] (Figure 2(a)) and [M(L)(OH_2_)_4_]Cl (Figure 2(b)) composition of the metal(II) chelates. Efforts to grow good crystals of the ligands and their metal chelates for X-ray diffraction studies were unsuccessful due to their poor solubility in common organic solvents.

### IR spectra

Diketones and related compounds such as acetylacetone in the present studies are capable of exhibiting keto-enol tautomerism and react with metal cations to form metal complexes. The selected IR spectra of the ligands and its metal(II) complexes along with their tentative assignments are reported in “experimental” and in Table 2, respectively. The IR spectra of all the ligands show [43] the absence of bands at 3245 and 1745 cm^−1^ due to *ν*(HN_2_) group of amino acids and *ν*(C=O) of acetylacetone. Instead, a new prominent band at 1635 cm^−1^ due to azomethine *ν*(C=N) linkage appeared in all the ligands indicating [44] that condensation between ketone moiety of acetylacetone and that of amino group of amino acid has taken place resulting into the formation of the desired ligands (L_1_)–(L_5_). Also, the presence of bands at 3015–3025 and 3444–3450 cm^−1^ due to *ν*(C=C) and *ν*(OH) in the ligands clearly gave an evidence [43] of establishing keto-enol tautomeric system in which these ligands behave as enol. Moreover, on comparison of the IR spectra of the ligands with their metal(II) complexes showed [45] a major shift to lower wave numbers by 15–20 cm^−1^ in azomethine *ν*(C=N) at 1610–1620 cm^−1^ suggesting involvement of the azomethine-N with the metal(II) ion. Also, disappearance of the stretching frequency at 1700–1708 cm^−1^ assigned to *ν*(COOH) and appearance of new *ν*_as_ and *ν*_s_ modes of the (−CO_2_) group at 1590 and 1385 cm^−1^, respectively, the Δ*ν* value (205 cm^−1^) is consistent with carboxylate coordination with the metal atoms. These overall data suggest that the azomethine-N and carboxylate-O groups are involved in coordination with the metal(II) ion in complexes (1)–(40). In the low-frequency region, spectra of the metal(II) complexes (Table 1) exhibited [46] new bands which are not present in the spectra of the ligands. These bands are located at 525 and 470 cm^−1^, which are attributed to *ν*(M−O) and *ν*(M−N). The coordinated water in all the metal(II) complexes presents different peaks at 990 cm^−1^ (rocking) and 760 cm^−1^ (wagging), whereas none of these vibrations appear in the spectra of uncoordinated ligands.

### NMR spectra

The ^1^H NMR spectral data are reported along with the possible assignments in “experimental.” All the protons were found as to be in their expected region [47]. The conclusions drawn from these studies lend further support to the mode of bonding discussed in their IR spectra. In the spectra of diamagnetic Zn(II) complexes, coordination of the ligands via azomethine-N and carboxylate-O was established by downfield shifting of these signals in the Zn(II) complexes due to the increased conjugation and coordination [48]. The number of protons calculated from the integration curves and those obtained from the values of the expected CHN analyses agree with each other. It was observed that DMSO did not have any coordinating effect neither on the spectra of the ligands nor on its metal complexes.

### Electronic spectra

The Co(II) complexes exhibited well-resolved bands at 17543–18018 cm^−1^ and a strong high-energy band at 21739–22222 cm^−1^ (Table 2) and are assigned [49] to the transitions ^4^T_1g_(F)→^4^T_2g_(F), ^4^T_1g_(F)→^4^T_1g_(P) for a high-spin octahedral geometry [50]. A high-intensity band at 28565–29215 cm^−1^ was assigned to the metal to ligand charge transfer. The magnetic susceptibility measurements (4.7–4.9 BM) for the solid Co(II) complexes are also indicative of three unpaired electrons per Co(II) ion suggesting [51] consistency with their octahedral environment. The electronic spectra of the Cu(II) complexes (Table 2) showed two low-energy weak bands at 15151–15873 cm^−1^ and a strong high-energy band at 30255–30420 cm^−1^. The low-energy band in this position typically is expected for an octahedral configuration and may be assigned to 10 Dq corresponding to the transition ^2^Eg→^2^T_2g_ [49]. The strong high-energy band, in turn, is assigned to metal → ligand charge transfer. Also, the magnetic moment values (1.9–2.2 BM) for the copper(II) are indicative of antiferromagnetic spin-spin interaction through molecular association. Hence, the copper(II) complexes appear to be in the octahedral geometry with d^2^_x_–d^2^_y_ ground state [51]. The electronic spectra of the Ni(II) complexes showed d-d bands in the regions 24390–25000, 16528–16667, and 12987–13333 cm^−1^. These are assigned to the spin-allowed transitions ^3^A_2g_(F)→^3^T_2g_(F), ^3^A_2g_(F)→^3^T_1g_(F), and ^3^A_2g_(F)→^3^T_1g_(P), respectively, consistent with their well-defined octahedral configuration. The band at 29815–30335 cm^−1^ was assigned to metal → ligand charge transfer. The magnetic measurements (3.0–3.3 BM) showed two unpaired electrons per Ni(II) ion suggesting [52] also an octahedral geometry for the Ni(II) complexes. The electronic spectra of the Zn(II) complexes exhibited only a high-intensity band at 28 350–29 145 cm^−1^ and are assigned [49] to a ligand-metal charge transfer.

### Biological activity

The antibacterial activity results presented in Table 3 show that the newly synthesized compounds (L_1_)–(L_5_) and their metal(II) complexes (1)–(40) possess biological activity. These new derivatives obtained by condensation of the amino group of amino acid with salicylaldehyde were screened for their antibacterial activity against *E coli, B subtillis, S flexenari, S aureus, P aeruginosa, and S typhi* and for antifungal activity (Table 4) against *T longifusus, C albicans, A flavus, M canis, F solani*, and *C glaberata*. These results exhibited markedly an enhancement in activity on coordination with the metal ions against one or more testing bacterial strains. This enhancement in the activity is rationalized on the basis of the structures of, (L_1_)–(L_5_) by possessing an additional azomethine (C=N) linkage which imports in elucidating the mechanism of transamination and resamination reactions in biological system [53, 54]. It has also been suggested [55–65] that the ligands with nitrogen and oxygen donor systems might inhibit enzyme production, since the enzymes which require these groups for their activity appear to be especially more susceptible to deactivation by the metal ions upon chelation. Chelation reduces the polarity [55–65] of the metal ion mainly because of the partial sharing of its positive charge with the donor groups and possibly the *π*-electron delocalization within the whole chelate ring system thus formed during coordination. This process of chelation thus increases the lipophilic nature of the central metal atom, which in turn favors its permeation through the lipoid layer of the membrane. This in turn is responsible for increasing the hydrophobic character and liposolubility of the molecule in crossing cell membrane of the microorganism, and hence enhances the biological utilization ratio and activity of the testing drug/compound.

### Cytotoxic bioassay

All the synthesized compounds were screened for their cytotoxicity (brine shrimp bioassay) using the protocol of Meyer et al [66]. From the data recorded in Table 6, it is evident that only five compounds (3), (7), (10), (11), and (22) displayed potent cytotoxic activity as LD_50_ = 8.974 × 10^−4^, 7.022 × 10^−4^, 8.839×10^−4^, 7.133×10^−4^, and 9.725×10^−4^ M/mL, respectively, against *Artemia salina* while all other compounds were almost inactive for this assay.

## CONCLUSION

The synthesized amino acid-derived compounds showed antibacterial/antifungal properties. In comparison, the cobalt (II), copper(II), nickel(II), and zinc(II) metal complexes of these compounds showed more activity against one or more bacterial/fungal strains, thus introducing a novel class of metal-based bactericidal and fungicidal agents.

## Figures and Tables

**Table 1:** Physical and analytical data of the metal(II) complexes (1)–(40).

Number	Metal chelate	MP (°C)	Yield (%)	Calc (found) %		
				C	H	N

(1)	[Co(L_1_)_2_(H_2_O)_2_] [406.9]	336–338	71	41.28 (41.61)	5.90 (5.42)	6.88 (6.13)
	C_14_H_24_CoN_2_O_8_					
(2)	[Cu(L_1_)_2_(H_2_O)_2_] [411.5]	328–330	73	40.82 (40.44)	5.83 (5.52)	6.80 (6.45)
	C_14_H_24_CuN_2_O_8_					
(3)	[Ni(L_1_)_2_(H_2_O)_2_] [406.7]	330–332	70	41.31 (41.65)	5.90 (5.98)	6.88 (6.57)
	C_14_H_24_NiN_2_O_8_					
(4)	[Zn(L_1_)_2_(H_2_O)_2_] [411.4]	331–332	70	40.84 (40.63)	5.83 (5.62)	6.81 (6.96)
	C_14_H_24_ZnN_2_O_8_					
(5)	[Co(L_2_)_2_(H_2_O)_2_] [586.9]	378–380	72	57.25 (57.53)	6.13 (6.55)	4.77 (4.63)
	C_28_H_36_CoN_2_O_8_					
(6)	[Cu(L_2_)_2_(H_2_O)_2_] [563.5]	335–337	72	56.80 (56.66)	6.09 (6.37)	4.73 (4.58)
	C_28_H_36_CuN_2_O_8_					
(7)	[Ni(L_2_)_2_(H_2_O)_2_] [586.7]	338–340	73	57.27 (57.14)	6.14 (6.47)	4.77 (4.84)
	C_28_H_36_NiN_2_O_8_					
(8)	[Zn(L_2_)_2_(H_2_O)_2_] [591.4]	332–334	72	56.82 (56.98)	6.09 (5.84)	4.73 (4.65)
	C_28_H_36_ZnN_2_O_8_					
(9)	[Co(L_3_)_2_(H_2_O)_2_] [490.9]	339–341	74	48.89 (48.73)	7.33 (7.62)	5.70 (5.53)
	C_20_H_36_CoN_2_O_8_					
(10)	[Cu(L_3_)_2_(H_2_O)_2_] [495.5]	344–346	73	48.43 (48.87)	7.26 (7.18)	5.65 (5.85)
	C_20_H_36_CuN_2_O_8_					
(11)	[Ni(L_3_)_2_(H_2_O)_2_] [490.7]	340–342	73	48.91 (48.76)	7.34 (7.58)	5.71 (5.43)
	C_20_H_36_NiN_2_O_8_					
(12)	[Zn(L_3_)_2_(H_2_O)_2_] [495.4]	337–339	72	48.45 (48.63)	7.27 (7.47)	5.65 (5.96)
	C_20_H_36_ZnN_2_O_8_					
(13)	[Co(L_4_)_2_(H_2_O)_2_] [566.9]	238–240	72	46.57 (46.66)	5.64 (5.53)	14.82 (14.72)
	C_22_H_32_CoN_6_O_8_					
(14)	[Cu(L_4_)_2_(H_2_O)_2_] [571.5]	230–232	70	46.19 (46.54)	5.60 (5.43)	14.70 (14.57)
	C_22_H_32_CuN_6_O_8_					
(15)	[Ni(L_4_)_2_(H_2_O)_2_] [566.7]	227–229	71	46.59 (46.62)	5.65 (5.57)	14.82 (14.66)
	C_22_H_32_NiN_6_O_8_					
(16)	[Zn(L_4_)_2_(H_2_O)_2_] [571.4]	225–227	72	46.20 (46.06)	5.60 (5.81)	14.70 (14.98)
	C_22_H_32_ZnN_6_O_8_					
(17)	[Co(L_5_)_2_(H_2_O)_2_] [434.9]	240–242	73	44.15 (44.48)	6.44 (6.16)	6.44 (6.82)
	C_16_H_28_CoN_2_O_8_					
(18)	[Cu(L_5_)_2_(H_2_O)_2_] [439.5]	244–246	72	43.68 (43.36)	6.37 (6.56)	6.37 (6.73)
	C_16_H_28_CuN_2_O_8_					
(19)	[Ni(L_5_)_2_(H_2_O)_2_] [434.7]	245–247	70	44.16 (44.44)	6.44 (6.38)	6.44 (6.16)
	C_16_H_28_NiN_2_O_8_					
(20)	[Zn(L_5_)_2_(H_2_O)_2_] [439.4]	236–238	69	43.70 (43.34)	6.37 (6.15)	6.37 (6.62)
	C_16_H_28_ZnN_2_O_8_					
(21)	[Co(L_1_)(H_2_O)_4_]Cl [322.4]	206–208	70	26.05 (26.37)	5.58 (5.41)	4.34 (4.13)
	C_7_H_18_CoNO_7_Cl					
(22)	[Cu(L_1_)(H_2_O)_4_]Cl [327.0]	216–218	71	25.68 (25.44)	5.50 (5.82)	4.28 (4.45)
	C_7_H_18_CuNO_7_Cl					
(23)	[Ni(L_1_)(H_2_O)_4_]Cl [322.2]	212–214	72	26.07 (26.38)	5.59 (5.88)	4.35 (4.54)
	C_7_H_18_NiNO_7_Cl					
(24)	[Zn(L_1_)(H_2_O)_4_]Cl [326.9]	202–204	70	25.70 (25.53)	5.51 (5.62)	4.28 (4.11)
	C_7_H_18_ZnNO_7_Cl					
(25)	[Co(L_2_)(H_2_O)_4_]Cl [412.4]	218–220	73	40.73 (40.93)	5.82 (5.55)	3.39 (3.18)
	C_14_H_24_CoNO_7_Cl					
(26)	[Cu(L_2_)(H_2_O)_4_]Cl [417]	227–229	72	40.28 (40.46)	5.75 (5.64)	3.36 (3.67)
	C_14_H_24_CuNO_7_Cl					
(27)	[Ni(L_2_)(H_2_O)_4_]Cl [412.2]	220–222	73	40.76 (40.43)	5.82 (5.64)	3.40 (3.13)
	C_14_H_24_NiNO_7_Cl					
(28)	[Zn(L_2_)(H_2_O)_4_]Cl [416.9]	214–216	72	40.30 (40.48)	5.76 (5.40)	3.36 (3.58)
	C_14_H_24_ZnNO_7_Cl					
(29)	[Co(L_3_)(H_2_O)_4_]Cl [364.4]	230–232	70	32.93 (32.67)	6.59 (6.35)	3.84 (3.53)
	C_10_H_24_CoNO_7_Cl					
(30)	[Cu(L_3_)(H_2_O)_4_]Cl [369.0]	238–240	71	32.52 (32.84)	6.50 (6.18)	3.79 (3.88)
	C_10_H_24_CuNO_7_Cl					
(31)	[Ni(L_3_)(H_2_O)_4_]Cl [364.2]	240–242	72	32.95 (33.28)	6.59 (6.34)	3.84 (3.63)
	C_10_H_24_NiNO_7_Cl					
(32)	[Zn(L_3_)(H_2_O)_4_]Cl [368.9]	235–237	73	32.53 (32.43)	6.51 (6.87)	3.80 (3.96)
	C_10_H_24_ZnNO_7_Cl					
(33)	[Co(L_4_)(H_2_O)_4_]Cl [402.4]	233–235	73	32.80 (32.66)	5.47 (5.53)	10.44 (10.72)
	C_11_H_22_CoN_3_O_7_Cl					
(34)	[Cu(L_4_)(H_2_O)_4_]Cl [407.0]	235–237	74	32.43 (32.64)	5.40 (5.27)	10.32 (10.57)
	C_11_H_22_CuN_3_O_7_Cl					
(35)	[Ni(L_4_)(H_2_O)_4_]Cl [402.2]	220–222	73	32.82 (32.58)	5.47 (5.65)	10.44 (10.68)
	C_11_H_22_NiN_3_O_7_Cl					
(36)	[Zn(L_4_)(H_2_O)_4_]Cl [406.9]	238–240	72	32.44 (32.06)	5.41 (5.83)	10.32 (10.78)
	C_11_H_22_ZnN_3_O_7_Cl					
(37)	[Co(L_5_)(H_2_O)_4_]Cl [336.4]	244–246	73	28.53 (28.68)	5.94 (5.64)	4.16 (4.52)
	C_8_H_20_CoNO_7_Cl					
(38)	[Cu(L_5_)(H_2_O)_4_]Cl [341.0]	248–250	72	28.15 (28.36)	5.86 (5.56)	4.11 (4.43)
	C_8_H_20_CuNO_7_Cl					
(39)	[Ni(L_5_)(H_2_O)_4_]Cl [336.2]	244–246	73	28.56 (28.74)	5.95 (5.78)	4.16 (4.56)
	C_8_H_20_NiNO_7_Cl					
(40)	[Zn(L_5_)(H_2_O)_4_]Cl [340.9]	247–249	72	28.16 (28.48)	5.87 (5.65)	4.11 (4.42)
	C_8_H_20_ZnNO_7_Cl					

**Table 2:** Physical and spectral data of the metal(II) complexes (1)–(40).

Number	Color	BM (*μ*_eff_)	IR ( cm^−1^)	*λ* max ( cm^−1^)

(1)	Dark brown	4.4	3444 (OH), 3020 (OH_2_),	17543, 21739, 29290
			1610 (C=N), 1385 (C−O),	
			525 (M−O), 470 (M−N)	
(2)	Light blue	1.7	3450 (OH), 3025 (OH_2_),	15151, 30235
			1620 (C=N), 1335 (C−O),	
			440 (M−N), 520 (M−O)	
(3)	Dull green	3.1	3445 (OH), 3015 (OH_2_),	12897, 16528, 24390, 30215
			1615 (C=N), 1335 (C−O),	
			430 (M−N), 535 (M−O)	
(4)	Off-white	Dia	3448 (OH), 3025 (OH_2_),	28445
			1610 (C=N), 1335 (C−O),	
			435 (M−N), 545 (M−O)	
(5)	Dark brown	4.2	3444 (OH), 3025 (OH_2_),	18018, 22222, 29565
			1615 (C=N), 1335 (C−O),	
			425 (M−O), 390 (M−N)	
(6)	Dark blue	1.7	3444 (OH), 3015 (OH_2_),	15873, 30380
			1615 (C=N), 1335 (C−O),	
			425 (M−O), 390 (M−N)	
(7)	Dark green	3.1	3448 (OH), 3020 (OH_2_),	13333, 16667, 25000, 30365
			1620 (C=N), 1335 (C−O),	
			425 (M−O), 390 (M−N)	
(8)	Cream	Dia	3445 (OH), 3020 (OH_2_),	28680
			1620 (C=N), 1335 (C−O),	
			425 (M−O), 390 (M−N)	
(9)	Brown	4.5	3448 (OH), 3025 (OH_2_),	17750, 21535, 29310
			1610 (C=N), 1335 (C−O),	
			425 (M−O), 390 (M−N)	
(10)	Bluish green	1.8	3450 (OH), 3015 (OH_2_),	15470, 30355
			1615 (C=N), 1335 (C−O),	
			425 (M−O), 390 (M−N)	
(11)	Dark green	3.3	3444 (OH), 3015 (OH_2_),	12975, 16585, 24685, 30310
			1610 (C=N), 1335 (C−O),	
			425 (M−O), 390 (M−N)	
(12)	Pale yellow	Dia	3450 (OH), 3020 (OH_2_),	28525
			1615 (C=N), 1335 (C−O),	
			425 (M−O), 390 (M−N)	
(13)	Tea pink	4.3	3445 (OH), 3015 (OH_2_),	17850, 21950, 29410
			1610 (C=N), 1335 (C−O),	
			425 (M−O), 390 (M−N)	
(14)	Green	1.9	3448 (OH), 3025 (OH_2_),	15510, 30290
			1615 (C=N), 1335 (C−O),	
			425 (M−O), 390 (M−N)	
(15)	Sea green	3.2	3445 (OH), 3025 (OH_2_),	13230, 16660, 24880, 30360
			1620 (C=N), 1335 (C−O),	
			425 (M−O), 390 (M−N)	
(16)	Off-white	Dia	3444 (OH), 3020 (OH_2_),	30360
			1615 (C=N), 1335 (C−O),	
			425 (M−O), 390 (M−N)	
(17)	Dark brown	4.5	3450 (OH), 3015 (OH_2_),	17985, 22125, 29490
			1620 (C=N), 1335 (C−O),	
			425 (M−O), 390 (M−N)	
(18)	Blue	1.8	3450 (OH), 3020 (OH_2_),	15750, 30360
			1620 (C=N), 1335 (C−O),	
			425 (M−O), 390 (M−N)	
(19)	Dark green	3.4	3444 (OH), 3020 (OH_2_),	13215, 16575, 24910, 30355
			1610 (C=N), 1335 (C−O),	
			425 (M−O), 390 (M−N)	
(20)	Cream	Dia	3445 (OH), 3020 (OH_2_),	28610
			1620 (C=N), 1335 (C−O),	
			425 (M−O), 390 (M−N)	
(21)	Dark blue	4.2	3450 (OH), 3025 (OH_2_),	18010, 21745, 29290
			1615 (C=N), 1335 (C−O),	
			425 (M−O), 390 (M−N)	
(22)	Green	1.7	3450 (OH), 3015 (OH_2_),	15545, 30235
			1610 (C=N), 1335 (C−O),	
			440 (M−N), 520 (M−O)	
(23)	Dirty green	3.1	3450 (OH), 3015 (OH_2_),	12897, 16580, 24490, 30215
			1615 (C=N), 1335 (C−O),	
			430 (M−N), 535 (M−O)	
(24)	Off-white	Dia	3450 (OH), 3025 (OH_2_),	28445
			1620 (C=N), 1335 (C−O),	
			435 (M−N), 545 (M−O)	
(25)	Dark blue	4.4	3448 (OH), 3020 (OH_2_),	17500, 22124, 29565
			1615 (C=N), 1335 (C−O),	
			425 (M−O), 390 (M−N)	
(26)	Dirty green	1.7	3450 (OH), 3025 (OH_2_),	15795, 30380
			1615 (C=N), 1335 (C−O),	
			425 (M−O), 390 (M−N)	
(27)	Sea green	3.1	3448 (OH), 3015 (OH_2_),	13233, 16590, 25000, 30365
			1615 (C=N), 1335 (C−O),	
			425 (M−O), 390 (M−N)	
(28)	Pale yellow	Dia	3450 (OH), 3020 (OH_2_),	28680
			1620 (C=N), 1335 (C−O),	
			425 (M−O), 390 (M−N)	
(29)	Royal blue	4.5	3450 (OH), 3025 (OH_2_),	17750, 21995, 29310
			1610 (C=N), 1335 (C−O),	
			425 (M−O), 390 (M−N)	
(30)	Green	1.8	3448 (OH), 3015 (OH_2_),	15490, 30355
			1620 (C=N), 1335 (C−O),	
			425 (M−O), 390 (M−N)	
(31)	Dull green	3.3	3448 (OH), 3020 (OH_2_),	12995, 16655, 24685, 30310
			1620 (C=N), 1335 (C−O),	
			425 (M−O), 390 (M−N)	
(32)	Yellow	Dia	3450 (OH), 3025 (OH_2_),	28525
			1615 (C=N), 1335 (C−O),	
			425 (M−O), 390 (M−N)	
(33)	Purple blue	4.3	3450 (OH), 3025 (OH_2_),	17855, 21925, 29410
			1610 (C=N), 1335 (C−O),	
			425 (M−O), 390 (M−N)	
(34)	Bluish green	1.9	3448 (OH), 3015 (OH_2_),	15515, 30290
			1620 (C=N), 1335 (C−O),	
			425 (M−O), 390 (M−N)	
(35)	Dirty green	3.2	3450 (OH), 3020 (OH_2_),	13130, 16565, 24880, 30360
			1620 (C=N), 1335 (C−O),	
			425 (M−O), 390 (M−N)	
(36)	Pale yellow	Dia	3450 (OH), 3025 (OH_2_),	30360
			1615 (C=N), 1335 (C−O),	
			425 (M−O), 390 (M−N)	
(37)	Dark brown	4.5	3448 (OH), 3015 (OH_2_),	17985, 22125, 29490
			1615 (C=N), 1335 (C−O),	
			425 (M−O), 390 (M−N)	
(38)	Green	1.8	3450 (OH), 3020 (OH_2_),	15750, 30360
			1620 (C=N), 1335 (C−O),	
			425 (M−O), 390 (M−N)	
(39)	Light green	3.4	3448 (OH), 3020 (OH_2_),	13215, 16570, 24910, 30355
			1610 (C=N), 1335 (C−O),	
			425 (M−O), 390 (M−N)	
(40)	Cream	Dia	3450 (OH), 3015 (OH_2_),	28610
			1620 (C=N), 1335 (C−O),	
			425 (M−O), 390 (M−N)	

**Table 3:** Results of antibacterial bioassay (concentration used 1 mg/mL of DMSO). (a) *E coli*, (b) *S flexenari*, (c) *P aeruginosa*, (d) *S typhi*, (e) *S aureus*, (f) *B subtilis* 10 <: weak; > 10: moderate; > 16: significant.

		Bacteria					
		
		Gram-negative				Gram-positive	

		(a)	(b)	(c)	(d)	(e)	(f)

Compound (zone of inhibition)	L_1_	12	07	13	11	16	15
	L_2_	14	07	14	14	15	16
	L_3_	14	08	12	15	16	17
	L_4_	13	05	14	14	17	14
	L_5_	12	07	15	15	17	15
	1	16	10	16	16	18	17
	2	15	11	15	17	18	18
	3	15	10	17	18	18	18
	4	16	12	22	18	19	19
	5	15	10	17	18	19	18
	6	15	10	16	17	19	17
	7	16	11	17	18	20	18
	8	16	11	18	19	21	19
	9	17	10	17	17	18	18
	10	16	10	18	16	19	19
	11	17	11	16	17	19	18
	12	19	12	17	24	20	19
	13	16	10	16	19	19	18
	14	16	11	17	17	17	18
	15	17	10	18	18	18	17
	16	18	11	17	20	20	20
	17	14	09	17	17	18	18
	18	17	10	18	18	19	19
	19	19	09	16	18	19	19
	20	25	10	19	18	20	21
	21	12	07	13	12	15	17
	22	11	06	14	13	16	18
	23	12	06	12	12	17	16
	24	15	09	16	14	18	24
	25	12	08	14	13	16	16
	26	12	07	15	12	15	17
	27	14	08	14	12	17	19
	28	15	09	16	14	18	19
	29	11	08	12	12	14	15
	30	12	07	12	11	16	16
	31	13	07	14	13	15	16
	32	14	10	15	15	17	18
	33	13	08	14	14	16	17
	34	14	09	13	15	15	16
	35	12	07	14	15	16	17
	36	14	11	16	17	17	18
	37	11	09	15	14	15	18
	38	12	08	15	15	16	16
	39	13	09	14	16	17	17
	40	15	10	16	17	26	19
	*SD	30	27	26	27	30	28

*SD: standard drug (Imipenem).

**Table 4:** Results of antifungal bioassay (concentration used 200 *μ*g/mL). (a) *T longifucus*, (b) *C albicans*, (c) *A flavus*, (d) *M canis*, (e) *F solani*, (f) *C glaberata*.

	Organism						
	
		(a)	(b)	(c)	(d)	(e)	(f)

Compound (zone of inhibition)	L_1_	16	00	15	10	00	18
	L_2_	00	07	00	00	15	00
	L_3_	17	00	00	00	00	00
	L_4_	20	00	00	15	00	20
	L_5_	00	00	00	00	00	00
	1	17	00	18	15	00	20
	2	18	00	20	14	00	18
	3	20	00	19	12	00	19
	4	22	00	20	21	00	22
	5	00	10	00	00	17	00
	6	10	17	00	00	18	17
	7	00	15	00	00	18	00
	8	00	18	00	00	20	00
	9	19	00	00	00	00	00
	10	20	00	17	00	00	00
	11	22	00	00	00	00	00
	12	24	00	00	00	00	00
	13	22	00	00	00	00	00
	14	24	20	00	25	20	20
	15	23	00	00	00	00	00
	16	25	00	18	30	00	00
	17	00	00	00	00	00	00
	18	00	00	00	00	00	00
	19	00	00	00	00	00	00
	20	00	00	00	00	00	00
	21	00	00	00	19	00	00
	22	00	00	00	00	00	00
	23	00	18	00	00	00	00
	24	20	00	00	00	24	18
	25	00	17	17	17	17	00
	26	00	00	15	00	00	17
	27	00	00	00	00	15	00
	28	00	00	00	00	00	00
	29	00	00	00	00	00	00
	30	00	00	00	00	00	00
	31	00	00	00	00	00	00
	32	00	20	00	19	00	00
	33	00	20	20	20	20	20
	34	00	00	00	00	00	20
	35	00	00	19	00	00	00
	36	00	00	00	00	00	00
	37	00	00	00	00	00	00
	38	00	00	00	00	00	00
	39	00	00	00	00	00	00
	40	00	00	19	00	00	20
	*SD	A	B	C	D	E	F

*SD = standard drugs MIC *μ*g/mL; A = Miconazole (70 *μ*g/mL: 1.6822 × 10^−7^ M), B = Miconazole (110.8 *μ*g/mL: 2.6626 × 10^−7^ M), C = Amphotericin B (20 *μ*g/mL: 2.1642×10^−8^ M), D=Miconazole (98.4 *μ*g/mL: 2.3647 × 10^−7^ M), E = Miconazole (73.25 *μ*g/mL: 1.7603 × 10^−7^ M), F = Miconazole (110.8 *μ*g/mL: 2.66266 × 10^−7^ M).

**Table 5:** Results of minimum inhibitory concentration (M/mL) of the selected compounds (4), (12), (20), (24), and (40) against selected bacteria.

Number	4	12	20	24	40

Gram-negative
*E coli*	—	—	5.690 × 10^−8^	—	—
*P aeruginosa*	1.215 × 10^−7^	—	—	—	—
*S typhi*	—	5.046 × 10^−8^	—	—	—

Gram-positive
*S aureus*	—	—	—	—	2.933 × 10^−8^
*B subtilis*	—	—	—	7.648 × 10^−8^	—

**Table 6:** Brine shrimp bioassay data of the ligands (L_1_)–(L_5_) and their metal(II) complexes (1)–(40).

Compound	LD_50_ (M/mL)

L_1_	6.369 × 10^−3^
L_2_	4.292 × 10^−3^
L_3_	5.025 × 10^−3^
L_4_	4.484 × 10^−3^
L_5_	5.848 × 10^−3^
1	2.458 × 10^−3^
2	2.430 × 10^−3^
3	8.975 × 10^−4^
4	2.431 × 10^−3^
5	1.704 × 10^−3^
6	1.691 × 10^−3^
7	7.022 × 10^−4^
8	1.691 × 10^−3^
9	2.037 × 10^−3^
10	8.839 × 10^−4^
11	7.133 × 10^−4^
12	2.018 × 10^−3^
13	1.764 × 10^−3^
14	1.750 × 10^−3^
15	1.765 × 10^−3^
16	1.750 × 10^−3^
17	2.299 × 10^−3^
18	2.275 × 10^−3^
19	2.300 × 10^−3^
20	2.276 × 10^−3^
21	3.102 × 10^−3^
22	9.725 × 10^−4^
23	3.104 × 10^−3^
24	3.059 × 10^−3^
25	2.425 × 10^−3^
26	2.398 × 10^−3^
27	2.426 × 10^−3^
28	2.399 × 10^−3^
29	2.744 × 10^−3^
30	2.710 × 10^−3^
31	1.112 × 10^−3^
32	2.711 × 10^−3^
33	2.485 × 10^−3^
34	2.457 × 10^−3^
35	2.486 × 10^−3^
36	2.458 × 10^−3^
37	2.973 × 10^−3^
38	1.246 × 10^−3^
39	2.974 × 10^−3^
40	2.933 × 10^−3^
